# Risk of Being Born Preterm in Offspring of Cancer Survivors: A National Cohort Study

**DOI:** 10.3389/fonc.2020.01352

**Published:** 2020-08-04

**Authors:** Wuqing Huang, Kristina Sundquist, Jan Sundquist, Jianguang Ji

**Affiliations:** ^1^Center for Primary Health Care Research, Lund University/Region Skåne, Malmö, Sweden; ^2^Department of Family Medicine and Community Health, Department of Population Health Science and Policy, Icahn School of Medicine at Mount Sinai, New York, NY, United States; ^3^Department of Functional Pathology, Center for Community-Based Healthcare Research and Education (CoHRE), School of Medicine, Shimane University, Matsue, Japan

**Keywords:** preterm birth, cancer, survivorship, offspring, epidemiology

## Abstract

**Background:** With the increased number of cancer survivors, it is necessary to explore the effect of cancer and its treatments on pregnancy outcomes, such as preterm birth, which seriously endangers the health of offspring. We aimed to explore the risk of being born preterm among offspring of cancer survivors.

**Materials and Methods:** This is a retrospective cohort study. All singleton live births between 1973 and 2014 in Sweden with information of birth outcomes were retrieved from the Swedish Medical Birth Register. By linking to several Swedish registers, we identified all parents of children and parental cancer diagnosis. Logistic regression was used to estimate odds ratios and 95% confidence intervals.

**Results:** As compared to the children without parental cancer, the risk of being born preterm was significantly higher among children of overall female cancer survivors born after cancer diagnosis with an adjusted OR of 1.48 (95 CI% = 1.39–1.59), in particular those diagnosed with childhood cancer and cancer in female genital organs. Besides, the risk might continuously decline with time at the first 8 years after maternal diagnosis. A higher risk of being born preterm was found among offspring of male survivors diagnosed with central nervous system cancer (Adjusted *OR* = 1.26, 95% CI = 1.04–1.53).

**Conclusions:** Our study provides evidence for a higher risk of being born preterm among children of female cancer survivors and male survivors with central nervous system tumor, as well as indicates that the effect on female reproductive system from cancer and related-treatments might decline with time.

## Background

With the great development of cancer therapies and cancer screening, the overall cancer survival rate has improved during recent decades. For example, in America, the statistical evidence reported in 2016 showed that over 15.5 million cancer survivors were alive on January 1, 2016 and the number is expected to increase to 20 million by January 1, 2026 (1); Globally, ~33 million individuals with a history of cancer had lived for over 5 years after the diagnosis in 2012 (2). This drives further studies about long-term effect or “late effect” caused by cancer itself and relevant treatments, including chemotherapy and radiotherapy (3). There is growing evidence that both females and males with a history of cancer were less likely to have a child compared to the general population, besides female survivors may suffer a higher risk of complications during pregnancy and adverse pregnancy outcomes (4–23). It indicates that cancer survivors are at an increased risk of disorders in the reproductive system.

Preterm birth (PTB) is the dominating cause of neonatal death with an estimated number of 1.1 million infants dying from complications of PTB each year (24). Furthermore, PTB is the second most common cause of death among children under 5 years of age around the world, and children with PTB suffer a higher risk of long-term growth damage and morbidity, such as neurologic and developmental disabilities (24). In 2008, the rates of PTB across Europe ranged from 5.5 to 11.1% for all live births and from 4.3 to 8.7% for singleton birth (25). In Sweden, the prevalence of PTB has been relatively stable during recent decades with an estimated rate of around 6%.

The objectives of our study are (1) to examine whether the risk of being born preterm is higher among offspring of cancer survivors as compared with offspring from healthy parents, (2) to explore whether the incidence of PTB might be negatively associated with the time interval between the diagnosis of parental cancer and the delivery of children based on the hypothesis that the adverse effect in the genital system and germ cells caused by chemotherapy or radiotherapy might be recovered gradually, and (3) to examine whether the observed effect on PTB is heterogeneous in relation to various cancer types.

## Materials and Methods

### Study Population

All singleton live births between 1973 and 2014 in Sweden were included in the Swedish Medical Birth Register. By linking to the Swedish Multiple Generation Register and Swedish Cancer Registry, we could identify the parents of these children and obtain information about cancer diagnosis for parents. The Swedish Medical Birth Register was founded in 1973, which consisted of information related to pregnancy and childbirth (26). The Swedish Multi-Generation Register was created in 1932 where all individuals were linked to their first-degree relatives. The Swedish Cancer Registry was created in 1958 and used the 7th version of the International Classification of Diseases (ICD) code to record cancer diagnosis during the study period. As it is compulsory for clinicians, pathologists, and cytologists to report all newly diagnosed cancers to the Swedish Cancer Register, this Register is estimated to cover 90% newly-diagnosed cancer cases in Sweden (27).

A total of 12,583 children were identified with a maternal cancer and delivered 1 year after maternal cancer diagnosis and 13,253 with a paternal cancer diagnosis, which were the targeted population in this study. All childbirths born within 1 year after parental cancer diagnosis were excluded to make sure that the child was conceived after maternal or paternal cancer diagnosis. A total of 35,07,481 children were born in parents without cancer history, which is the reference group in the current study.

To control unmeasured confounding factors, two types of co-sibling analyses were further performed to compare differentially exposed siblings. The first one was to examine PTB in children, who were delivered after the diagnosis of cancer in their parents, and compared with their siblings who were delivered before the diagnosis of parental cancer. This analysis aimed to control unmeasured confounding factors shared in the siblings. Of targeted children of female survivors, 2,027 pairs of siblings were used in the first co-sibling study. It should be noted that female survivors might have multiple childbirths both before and after the diagnosis of cancer, thus these 2,027 pairs include 2,520 children born before maternal cancer diagnosis and 3,002 siblings born after maternal cancer diagnosis. Among offspring of male survivors, a total of 2,227 pairs of siblings were used in the this co-sibling study, including 3,765 children born before paternal cancer diagnosis and 4,723 siblings born after paternal cancer diagnosis. The second one was to examine PTB among children of cancer survivors who have more than one child after their diagnosis of cancer, and using the first child after parental cancer as the reference. This analysis aimed to explore whether PTB might be negatively associated with the time interval between the diagnosis of parental cancer and delivery of children. A total of 3,673 pairs in female survivors and 3,737 pairs in male survivors were used in the second co-sibling study.

The Ethics Committee at Lund University approved (February 6, 2013) this nationwide cohort study (Dnr 2012/795). Through advertisements in the major newspapers people could choose to opt out before the project database were constructed.

### Study Outcomes

Preterm birth was defined as a live birth occurring at less than 37 full weeks (<37 weeks) of gestation, early preterm birth as less than 32 weeks (<32 weeks) and extremely preterm birth as less than 28 weeks (<28 weeks) (24). Gestational age at birth was calculated by maternal report of last menstrual period in the 1970s and ultrasound estimation in the 1980s and later in Swedish Medical Birth Registry.

### Independent Variables

As shown in Table 1, the independent variables included child gender, year of childbirth, maternal age at birth, paternal age at birth, maternal age at diagnosis of cancer, paternal age at diagnosis of cancer, and the types of cancer in parents.

**Table 1:** Sociodemographic and clinical characteristics among offspring of female and male cancer survivors and controls.

**Variables**	**Offspring of female** **cancer survivors**		**Offspring of male** **cancer survivors**		**Offspring of parents both** **without cancer history**	
	**No. of** **individuals**	**No. of preterm** **birth, *N* (%)**	**No. of** **individuals**	**No. of preterm** **birth, *N* (%)**	**No. of** **individuals**	**No. of preterm** **birth, *N* (%)**
**Overall**	12,583	913 (7.26)	13,253	672 (5.07)	35,07,481	1,76,072 (5.02)
**Gender of offspring**						
Male	6,509	456 (7.01)	6,843	347 (5.07)	18,03,060	96,107 (5.33)
Female	6,074	457 (7.52)	6,410	325 (5.07)	17,04,421	79,965 (4.69)
**Year of childbirth**						
<1990	3,289	220 (6.69)	3,192	175 (5.48)	12,65,651	66,143 (5.23)
≥1990	9,294	693 (7.46)	10,061	497 (4.94)	22,41,830	1,09,929 (4.90)
**Maternal age at birth**						
<30	4,039	313 (7.75)	4,831	242 (5.01)	19,33,283	98,389 (5.09)
30–34	4,621	311 (6.73)	4,712	214 (4.54)	10,33,517	47,893 (4.63)
≥35	3,923	289 (7.37)	3,710	216 (5.82)	5,40,681	29,790 (5.51)
**Paternal age at birth**						
<30	4,107	297 (7.23)	2,474	119 (4.81)	13,24,997	70,502 (5.32)
30–34	4,107	297 (7.23)	4,090	205 (5.01)	11,48,367	53,470 (4.66)
≥35	5,531	368 (6.65)	6,689	348 (5.20)	10,34,117	52,100 (5.04)
**Age at diagnosis of cancer**						
Childhood cancer (0–14)	1,956	154 (7.87)	1,516	81 (5.34)		
Adolescence and young adult cancer (15–29)	8,130	562 (6.91)	7,413	374 (5.05)		
Adult cancer (>29)	2,497	197 (7.89)	4,324	217 (5.02)		
**Types of cancer**						
Digestive system	902	48 (5.32)	1,160	63 (5.43)		
Central nervous system	1,450	112 (7.72)	1,756	109 (6.21)		
Hematological malignancy	1,982	143 (7.21)	2,438	118 (4.84)		
Skin cancer and melanoma	2,635	163 (6.19)	1,948	106 (5.44)		
Male genital organs	–	–	3,069	147 (4.79)		
Female genital organs	2,108	242 (11.48)	–	–		
Others	4,121	260 (6.31)	3,089	140 (4.53)		

Child gender was modeled as either male or female. Year of child birth was modeled as <1990 or ≥1990. Age of parents at birth was modeled as <25, 25–29, and ≥30 years. Age at diagnosis of cancer in the parents was modeled as childhood cancer (age 0–14), adolescent and young adult cancer (age 15–29) and adult cancer (age >29). Cancer diagnosis was categorized into cancers in the digestive system (including upper aerodigestive tract, esophagus, salivary gland, stomach, small intestine, colon, rectum, anus, liver, and pancreas), hematological malignancy (including non-Hodgkin's lymphoma, Hodgkin's disease, myeloma, and leukemia), cancers in the male genital organs (including prostate, testis, and other male genital), cancers in the female genital organs (including breast, cervix, ovary, endometrium, uterus, and other female genital), and other cancers (including nose, lung, eye, breast, thyroid gland, and endocrine glands, bladder, kidney, bone, and connective tissue).

### Statistical Analysis

Unconditional logistic regression was used to estimate odds ratios (OR) and 95% confidence intervals (95% CI) for the association between PTB and parental cancer diagnosis by using offspring without parental cancer history as the reference category. Analyses were further stratified by gender of offspring, age at diagnosis of cancer in parents, year of childbirth and types of cancer in the parents. Given that *in-vitro* fertilization was first adopted in 1982 and very rare before 1990 in Sweden (28), stratified analysis was performed to see whether the risk of PTB among offspring of cancer survivors might be varied for those born before 1990 and those after 1990. Conditional logistic regression was used for co-sibling study design to estimate OR and 95% CI for the association between PTB and paternal cancer diagnosis. Both analyses were conducted, firstly unadjusted, and then adjusted for year of childbirth and parental age at birth.

Besides, multivariate logistic regression using restricted cubic splines with 4 knots was built to investigate how the impact on PTB risk changed with the increase of time interval between parental cancer diagnosis and childbirth among children of cancer survivors, adjusting for year of childbirth and parental age at birth.

Logistic regression analyses were performed using SAS version 9.3 (SAS Institute, Cary, NC), and restricted cubic splines analysis was performed in R package.

## Results

We present the basic characteristics of children of cancer survivors and the controls in Table 1. As shown in Table 1, a total of 913 (7.26%) PTBs were noted from 12,583 offspring of female cancer survivors, and 672 (5.07%) PTBs from 13,253 offspring of male cancer survivors. As for the controls, 1,76,072 (5.02%) PTBs were identified from 35,07,481 children.

**Figure 1:**
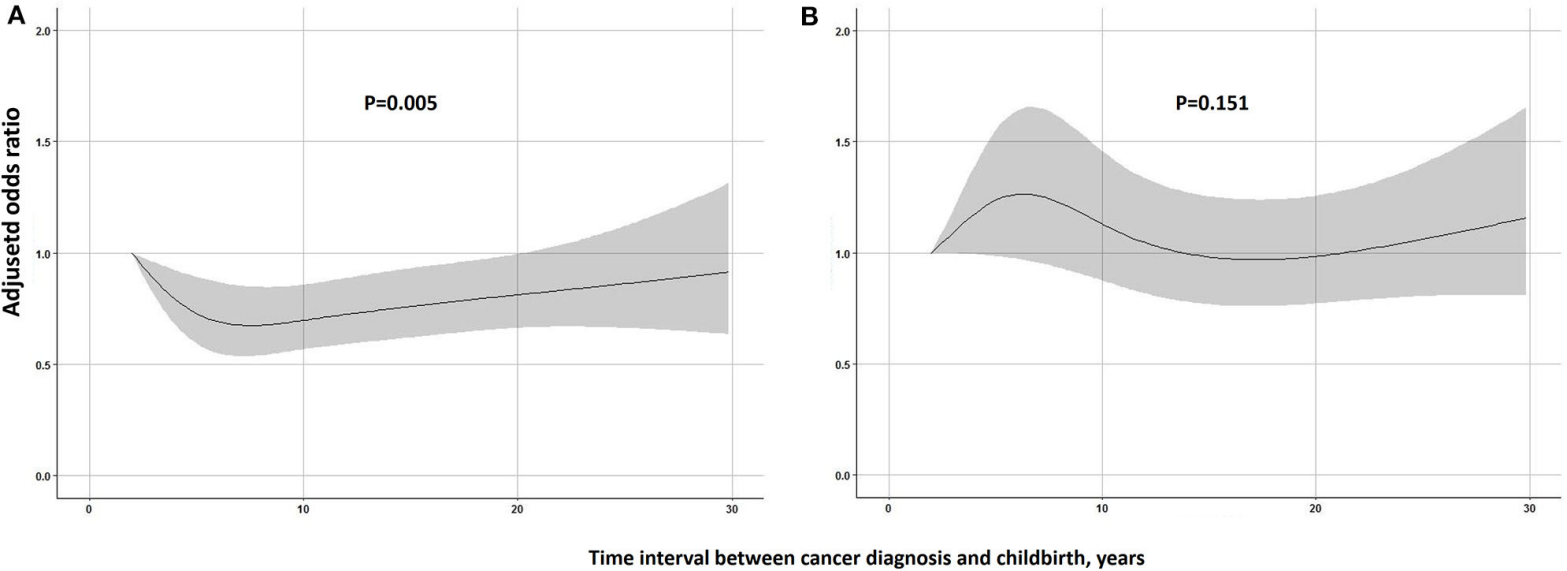
Restricted cubic spline modeling of the relationship of time interval parental between cancer diagnosis and childbirth with the risk of being born preterm in children. **(A)** Female cancer survivors. **(B)** Male cancer survivors. Adjusted odds ratios are shown as solid lines and 95% CIs as shaded areas (adjusted for year of childbirth, age of father at birth and age of mother at birth). Reference point is the lowest value of time interval.

Children born after maternal cancer diagnosis suffered a significantly increased risk of PTB when compared with the reference group (Table 2). The positive association remained similar after adjusting for year of childbirth and parental age at birth with an adjusted OR of 1.48 (95% CI 1.39–1.59). The association was slightly stronger in girls or children born after 1990. The risk of PTB was highest among children whose mothers were diagnosed with childhood cancer (Adjusted *OR* = 1.64, 95% CI 1.39–1.93), and the risk was 1.43 (95% CI 1.31–1.56) for children whose mothers were diagnosed with adolescent and young adult cancer and 1.53 (95% CI 1.33–1.77) for children whose mothers were diagnosed with adult cancer. Except for maternal cancers in the digestive system, maternal diagnosis with other types of cancer was found to be related to the risk of offspring's PTB, especially survivors with maternal diagnosis of female genital cancer (Adjusted *OR* = 2.42, 95% CI = 2.12–2.77). A greater risk of very preterm and extremely preterm births was also found among offspring of female cancer survivors when compared to the controls (Supplementary Table 1). No significant association of PTB risk was observed with children of male cancer survivors. When the analyses were stratified by cancer sites in the father, a significantly increased OR was found among children whose fathers were diagnosed with central nervous system cancer (Adjusted *OR* = 1.26, 95% CI 1.04–1.53). As shown in Figure 1, among children of overall female survivors, the adjusted OR significantly decreased with the increase of time interval at the first 8 years after maternal diagnosis and then slightly increased in the following years (*P*-value for non-linear = 0.005). But no significant non-linear or linear relationship was found in children of male survivors (*P*-value for non-linear = 0.151).

**Table 2:** Odd ratios (ORs) and 95% confidence intervals (CIs) of preterm birth among offspring of female and male cancer survivors compared with controls.

**Variables**	**Female cancer survivors**				**Male cancer survivors**			
	**Crude OR**	**95%CI**	**Adjusted OR^**a**^**	**95%CI**	**Crude OR**	**95%CI**	**Adjusted OR^**a**^**	**95%CI**
**Overall**	1.48	1.39–1.59	1.48	1.39–1.59	1.01	0.94–1.09	1.02	0.94–1.10
**Gender of offspring**								
Male	1.34	1.22–1.47	1.34	1.22–1.48	0.95	0.85–1.06	0.96	0.86–1.07
Female	1.65	1.50–1.82	1.64	1.49–1.81	1.09	0.97–1.22	1.09	0.97–1.22
**Year of childbirth**								
<1990	1.36	1.18–1.56	1.32	1.15–1.51	1.10	0.95–1.28	1.07	0.92–1.25
≥1990	1.53	1.42–1.65	1.54	1.42–1.66	0.98	0.90–1.08	1.00	0.92–1.10
**Age at diagnosis of cancer**								
Childhood cancer (0–14)	1.63	1.38–1.92	1.64	1.39–1.93	1.07	0.86–1.34	1.08	0.87–1.35
Adolescence and young adult cancer (15–29)	1.41	1.29–1.53	1.43	1.31–1.56	1.00	0.91–1.12	1.02	0.92–1.14
Adult cancer (>29)	1.63	1.41–1.89	1.53	1.33–1.77	1.00	0.87–1.15	0.99	0.86–1.13
**Types of cancer**								
Digestive system	1.07	0.80–1.42	1.06	0.79–1.42	1.09	0.85–1.41	1.09	0.84–1.40
Central nervous system	1.59	1.31–1.93	1.60	1.32–1.94	1.25	1.03–1.52	1.26	1.04–1.53
Hematological system	1.48	1.24–1.75	1.50	1.27–1.78	0.96	0.80–1.16	0.97	0.81–1.17
Skin cancer and melanoma	1.25	1.07–1.46	1.24	1.06–1.46	1.09	0.90–1.33	1.09	0.90–1.33
Male genital organs	–	–	–	–	0.95	0.81–1.13	0.97	0.82–1.14
Female genital organs	2.45	2.15–2.81	2.42	2.12–2.77	–	–	–	–
Others	1.28	1.12–1.45	1.26	1.11–1.43	0.90	0.76–1.07	0.90	0.76–1.07

a *Adjusted for year of childbirth, age of father at birth, and age of mother at birth*.

We present ORs and 95% CI of PTB using co-sibling design in Table 3. After adjusting for potential confounders, no significant association was found between maternal or paternal cancer diagnosis and PTB risk when comparing siblings born before and after cancer diagnosis. For families with more than one childbirth after parental cancer, we found that the risk of PTB in the children born later was significantly lower as compared with the first child. The adjusted OR was 0.49 for children of female survivors (95% CI 0.36–0.67) and 0.49 for male survivors (95% CI 0.35–0.67).

**Table 3:** Odd ratios (ORs) and 95% confidence intervals (CIs) of preterm birth among female and male cancer survivors using co-sibling design.

**Variables**	**No. of individuals**	**No. of preterm birth, *N* (%)**	**Crude OR**	**95%CI**	**Adjusted OR^**a**^**	**95%CI**
**Female cancer survivors**						
**Comparison between siblings born before and after diagnosis**						
Offspring born before diagnosis	3,002	174 (5.80)	1.00	–	1.00	–
Offspring born after diagnosis	2,520	192 (7.62)	1.43	1.12–1.83	1.24	0.79–1.94
**Comparison between children after diagnosis**						
First child	3,673	284 (7.73)	1.00	–	1.00	–
Second or more	4,698	269 (5.64)	0.63	0.52–0.78	0.49	0.36–0.67
**Male cancer survivors**						
**Comparison between siblings born before and after diagnosis**						
Offspring born before diagnosis	4,723	241 (5.10)	1.00	–	1.00	–
Offspring born after diagnosis	3,765	186 (4.94)	0.82	0.63–1.07	0.74	0.46–1.17
**Comparison between children after diagnosis**						
First child	3,737	213 (5.70)	1.00	–	1.00	–
Second or more	4,859	188 (3.87)	0.62	0.49–0.77	0.49	0.35–0.67

a *Adjusted for year of childbirth, age of father at birth, and age of mother at birth*.

## Discussion

In this retrospective cohort study, which is to our knowledge the largest study on this topic so far, we found that the risk of PTB was significantly higher among children of female cancer survivors born after maternal cancer diagnosis, and the risk was even more predominant for mothers with a childhood cancer and with cancers in the female genital organs. Although the overall risk of PTB was similar among children of male cancer survivors as compared to the controls, the risk was significantly increased among children whose fathers were diagnosed with tumors in the nervous system. When compared with the first childbirth of survivors, the risk of PTB was significantly decreased among their siblings born later. Besides, in female survivors, we found the risk continuously declined at the first 8 years after diagnosis, suggesting that the adverse effect on the reproductive system might be recovered gradually. It is thus of high clinical relevance for those cancer survivors who plan to have a child.

Recently, a growing number of studies estimated the risk of PTB among children of female cancer survivors, which concurs with our results that children with maternal cancer diagnosis were at an increased risk of PTB but differed in terms of maternal cancer types (3–6, 11, 14, 19, 21, 29, 30). Evidence from Cancer Registries in three U.S. states found a higher risk of PTB among first child of female survivors diagnosed with cervical, invasive breast cancer and leukemia, but no association with brain, thyroid cancer, melanoma, and Hodgkin lymphoma (21). The data were consistent with our results and found a higher PTB risk associated with maternal cancer in the female genital organs. In addition, our study found an elevated risk of PTB irrespective of the age at diagnosis of maternal cancer, and the risk was even higher in childhood cancer survivors. Concordant with our study, the nationwide study in Finland found that children of childhood cancer survivors had a 62% increased risk and children of young adult cancer survivors had a 36% increased risk compared with children of maternal siblings (11). A review targeting children of childhood, adolescent and young adult female cancer survivors showed that the rate of PTB was 1.5- to 2-fold higher in survivors compared with siblings or the general population (31), which suggests that age at diagnosis of maternal cancer had a different effect on the risk of PTB in their children. When we used co-sibling study design and compared with their siblings born before maternal cancer diagnosis, the association was not significant, suggesting that unmeasured familial factors might contribute to the observed association. However, we cannot exclude the possibility of false-negative due to a limited number of sibling pairs leading to a wide confidence interval.

Plenty of evidence suggests that cancer treatments, including chemotherapy drugs, and/or radiation, have a detrimental effect on ovary and uterus, and malfunction of these organs plays a key role in developing PTB (32). Among the female reproductive organs, the ovary has been found to be most sensitive to chemotherapy and was also compromised by radiation (32). Previous studies summarized the adverse outcomes of cancer treatments as either acute ovarian failure or premature ovarian failure (32). The former one damages the growing follicles, which is temporary and reversible, and more frequently diagnosed among cancer survivors diagnosed at an older age (33). The latter form may develop among some childhood cancer survivors who retain some ovarian function for a period and then experience a gradual irreversible diminished ovary function (8). In addition, majority of childhood cancer survivors tended to give a birth after over 15 years from cancer diagnosis which might be the reason why offspring of childhood cancer survivors had a higher risk of PTB. Besides, animal studies also observed that radiosensitivity of ovarian follicles differs depending upon developmental stages (34). The uterus does not seem to be affected by chemotherapy drugs according to the current evidence, while uterine function was significantly damaged by radiation therapy through influencing the endometrium, myometrium, and vascular structures in the uterus, especially when cancer survivors were exposed to pelvic, spinal, and abdominal irradiation, which would be responsible for the higher risk of PTB among children of female reproductive system cancer (31).

The current study found no association between paternal cancer diagnosis and overall offspring's PTB risk. Three previous studies, targeting on first offspring among male cancer survivors, found no significant increased risk as well (6, 14, 17). All of the previous studies were limited to Norway population and had partly overlapping data, additionally, the sample size was relatively smaller as compared to our study (6, 14, 17). In this study, it was noteworthy that an elevated risk was found among offspring of male survivors diagnosed as central nervous system tumor, which was in line with our previous study in survivors of childhood or adolescent central nervous system tumor (35). It is interesting that evidence from National Cancer Institute's Surveillance, Epidemiology, and End Results (SEER) Program suggested that female partners of male central nervous system cancer survivors tended to suffer with preeclampsia during a pregnancy that was associated with PTB (7, 36). Cranial irradiation is used alone or in combination with surgery and/or chemotherapy for central nervous system cancer which is able to affect hypothalamic-pituitary- adrenal and -gonadal axis that contributes to the decline of spermatogenetic quality (37, 38). Thus, the biological plausibility of paternal history with central nervous system cancer as a risk factor for preterm birth might be inferred through pregnancy complications or hypothalamic-pituitary- adrenal and -gonadal axis.

It is worth mentioning that in female survivors, we found a significant higher risk of PTB among first child born after cancer diagnosis as compared to siblings born later. Besides, restricted cubic splines regression allowed us to flexibly model and visualize the relationship and found that the risk of being born preterm in offspring of female survivors continuously declined in the first 8 years after parental cancer diagnosis. Such data strongly supports our hypothesis that damages to the female genital system due to chemo- or radiotherapy might be recovered gradually. For male survivors, their children were not at a significant higher risk of being born preterm when compared to general population and no significant variation of the association was observed with time, but a lower risk was observed among second child or more when compared with the first child. It might, to some extent, be related to “healthy worker effect,” i.e., only those male survivors who had a relatively good condition tended to have more than one child.

Some strengths of our study could be noted for this study. Firstly, to the best of our knowledge, this is the population-based study with largest sample size to investigate the adverse outcome in offspring of cancer survivors, allowing us to examine the continuous association modeling by restricted cubic splines regression. Secondly, Swedish Multi-Generation Register makes co-sibling design available which helps to exclude residual confounding by unmeasured environmental and/or genetic factors shared by the siblings. Besides, parental age at pregnancy can be adjusted in current study, which is an important factor for risk of adverse pregnancy outcomes. Finally, compared to self-reported data, our study enabled to avoid recall bias by using register-based data. However, lack of information about detailed cancer treatment made it unavailable to explore the specific impact of different cancer treatment on pregnancy outcomes. Besides we did not get access to the information about *in-vitro* fertilization, which might affect the risk of PTB. Stratified analysis by birth year before 1990 or after 1990 suggested that *in-vitro* fertilization might play a small role for the observed association.

The present study found an increased risk of being born preterm among children of female cancer survivors, in particular among children whose mother was diagnosed with childhood cancer and cancer in the female genital organs. In addition, paternal cancer in the central nervous system was a risk factor of offspring's PTB. Notably, the risk of being born preterm might decline with time within the first 8 years after diagnosis in children of female survivors. Our findings underline the necessity for continued prenatal follow-up of pregnancies among female cancer survivors and spouses of male survivors with central nervous system tumor. They are highly recommended to care about the timing of having a child to minimize the impacts caused by cancer and its therapy.

## Data Availability Statement

The datasets analyzed in this article are not publicly available. Registry data used for the analysis presented in this study are available from the appropriate Swedish authorities [the Swedish National Board of Health and Welfare (https://www.socialstyrelsen.se/en) and Statistics Sweden (https://www.scb.se/en)], for researchers who meet the criteria for access to confidential data.

## Ethics Statement

The studies involving human participants were reviewed and approved by the Ethics Committee at Lund University (Dnr 2012/795). Written informed consent was not needed in this register-based study as all individual identification information was removed to preserve anonymity.

## Author Contributions

WH, JJ, KS, and JS were responsible for the study concept and design. JS, KS, and JJ obtained funding. KS and JS acquired the data. WH did the statistical analysis and drafted the manuscript. All authors revised it for important intellectual content.

## Conflict of Interest

The authors declare that the research was conducted in the absence of any commercial or financial relationships that could be construed as a potential conflict of interest.
