# Supplementary material for: Risk of Being Born Preterm in Offspring of Cancer Survivors: A National Cohort Study
Source: Front Oncol. 2020 Aug 4;10:1352. doi: 10.3389/fonc.2020.01352 (PMC7418466; doi:10.3389/fonc.2020.01352)
Supplement: Supplementary file 1 [file Table_1.PDF]

Supplemental table 1 Odd ratios (ORs) and 95% confidence intervals (CIs) of very or extremely preterm birth (Gestational weeks<28 or 32) among children of female and male cancer survivors compared with offspring of parents both without cancer history

Variables	No of individuals	No of very preterm birth, N(%)	Crude OR	95%CI	Adjusted OR[a]	95%CI
**Female cancer survivors**						
Very preterm	11848	178(1.50)	1.87	1.61-2.17	1.86	1.60-2.16
Extremely preterm	11745	75(0.64)	1.76	1.40-2.21	1.80	1.43-2.26
**Male cancer survivors**						
Very preterm	12677	96(0.76)	0.94	0.77-1.15	0.94	0.77-1.16
Extremely preterm	13253	37(0.28)	0.81	0.58-1.11	0.86	0.62-1.18

[a] Adjusted for year of childbirth and parental age at birth.
